# Maximising Refractive Outcomes with an Extended Depth of Focus IOL

**DOI:** 10.2174/1874364101812010273

**Published:** 2018-09-28

**Authors:** Barry Power, Rory Murphy, Antonio Leccisotti, Tara Moore, William Power, Paul O’Brien

**Affiliations:** 1 Department of Ophthalmology, Mater Misericordiae University Hospital, Dublin, Ireland; 2 Department of Ophthalmology, Biomedical Sciences Research Institute, Ulster University, Northern Ireland; 3 Department of Ophthalmology, University of Siena, Siena, Italy; 4 Blackrock Clinic, Dublin, Ireland

**Keywords:** Cataract surgery, Corneal astigmatism, Refractive surgery, Symfony IOL, Extended depth of focus IOL, SIA

## Abstract

**Objective:**

To assess the impact of the magnitude of preoperative and postoperative corneal astigmatism on refractive outcomes in patients undergoing cataract surgery or lens exchange with an extended depth of focus intraocular lens. To compare visual outcomes of steep and temporal on-axis corneal incisions.

**Setting:**

Department of Ophthalmology, Blackrock Clinic, Dublin, Ireland.

**Design:**

Prospective cohort analysis.

**Methods:**

Fifty-three consecutive adult patients (94 eyes) undergoing routine phacoemulsification with Symfony IOL implantation were analysed. Exclusion criteria: targets for mini-monovision, incomplete data, other ocular pathology. Data were prospectively collected on pre- and postoperative refraction, keratometry, distance vision, near vision, surgical wound site and Surgically Induced Astigmatism (SIA).

**Results:**

The average postoperative monocular Uncorrected Distance and Near visual acuities (UDVA and UNVA) were 0.12 LogMAR (± 0.1) (6/7.5^+1^) and 0.34 LogMAR (± 0.09) respectively. The average binocular UDVA and UNVA were 0.05 (± 0.07) and 0.29 LogMAR (± 0.06) respectively. Low levels of preoperative corneal astigmatism (0-0.99 D) were associated with better LogMAR UDVA and UNVA when compared with higher levels (> 0.99 D): 0.11 (CI 0.103-0.107)* vs. *0.206 (CI 0.122-0.290) (*p* =0.015, CI 95%) and 0.33 (CI 0.316 - 0.356)* vs. *0.39 (CI 0.34-0.43) (*p* =0.034, CI 95%) respectively. When patients with steep on-axis corneal incisions were compared with temporal on-axis corneal incisions, no difference was detected in visual outcome or SIA.

**Conclusion:**

The Symfony IOL is an effective surgical means of addressing presbyopia and reducing postoperative spectacle dependence. We stress caution when offering potential spectacle independence for patients with over 1D of preoperative corneal astigmatism as these patients achieve statistically significantly inferior and less predictable visual results.

## 1 INTRODUCTION

Monofocal IOL insertion after phacoemulsification is a well-established surgical technique with exceptionally high patient satisfaction rates. However, it fails to address presbyopia. Presbyopia is associated with a decreased quality of life and patients are increasingly seeking partial or complete spectacle independence when undergoing cataract surgery [1]. A consensus on the optimum form of surgical presbyopic correction has not been reached.

A number of potential options exist. Over the past fifteen years, the most common options offered for the surgical correction of presbyopia have been monofocal IOL insertion with monovision and multifocal IOL insertion. Both techniques have advantages and disadvantages.

Monovison is almost 3 times as popular as multifocal IOL insertion, mainly due to physician and patient concern over photopic disturbances associated with multifocal IOLs. These photopic disturbances are well documented in clinical studies [2-4]. Monovision is not suitable for all patients as it requires a period of neuroadaptation, which some find difficult. It also impairs stereopsis [5]. For patients who wish to have binocular distance, intermediate and near vision two potential options exist: multifocal IOLs or the newly developed Extended Depth of Focus (EDF) IOLs.

Whereas multifocal IOLs have different foci for different points of focus, the EDF IOLs elongate a single focal point. The developers of these lenses believe that they can provide a similar vision to multifocal IOLs but with less debilitating symptoms of photopsia. In 2016, the Symfony IOL was the first EDF IOL to be approved by the FDA and it is increasingly popular. Hamid A *et al*. compared the Symfony IOL to two multifocal IOLs and found that the Symfony performed best for UDVA and UIVA but was inferior for UNVA [6]. The study also reported significantly fewer dysphotopsia complaints in the Symfony IOL group. Twenty percent of patients with multifocal IOLs complained of disturbing or troublesome haloes or glare compared with 5.6% of Symfony patients.

This lower rate of dysphotopsia in the Symfony lens compared with trifocal IOLs has not been reproduced by all studies; however Monaco *et al*. in a 2017 study of 152 eyes compared the Symfony IOL with a monofocal IOL (SN60WF) and a trifocal IOL (Panoptix) [7]. They found the trifocal lens to be superior to the Symfony IOL in UNVA with similar reported levels of dysphotopsia. Both were superior to the monofocal lens for near and intermediate vision with no difference in distance visual acuity.

The aim of each of these techniques is to maximise independence from spectacles. Attainment of complete or near-complete spectacle independence is affected by the degree corneal astigmatism. In this study, we aimed to assess the impact of the magnitude of pre- and postoperative corneal astigmatism on visual outcomes. We also wished to compare the effects of steep and temporal on axis corneal incisions on surgically induced astigmatism and visual outcomes. In doing so, we hoped to be able to produce data that would help us to select appropriate surgical candidates for EDF IOL insertion.

## 2 MATERIALS AND METHODS

Ethical approval was obtained from The Local Ethics Research Committee at Ulster University and the study adhered to the Tenets of The Declaration of Helsinki. Pre- and postoperative data were collected on 53 consecutive patients (94 eyes) who underwent uncomplicated phacoemulsification and Symfony IOL insertion over a 12-month period from September 2016 to September 2017. Data collected included preoperative Corrected Distance Visual Acuity (CDVA), refraction, keratometry and postoperative unaided distance (UDVA), near visual acuity (UNVA), refraction and keratometry. All preoperative visual acuities are best corrected and postoperative acuities are unaided. We also recorded the surgical wound site as either superior or temporal. All incisions were clear corneal incisions with a width of 2.4 mm. Mean follow up time was 4.1 months.

Two consultant surgeons performed all cases under topical anaesthesia using proxymetacaine and 1% intracameral lidocaine. Clear corneal incisions were performed using a 2.4 mm keratome. Nuclear fragmentation and removal were achieved via the divide and conquer technique or a modified chop technique. Symfony IOL insertion was performed using the DK7786 inserter.

Preoperative astigmatism was calculated using K values obtained from the IOL Master version 700. Distance vision was recorded in Snellen metres and converted to LogMAR for statistical analysis. Near vision was recorded at 40cm using the Jaeger reading cards. Surgically induced astigmatism was calculated with the Insight Eye Clinic online surgically induced astigmatism calculator. Statistical analysis between all subgroups was performed using the Mann-Whitney test with confidence intervals set to 95%. The two-tailed test was utilised.

Patients included were adults undergoing either refractive clear lens exchange or cataract surgery. Patient identifiers were removed from pre and postoperative data before data analysis. The range of preoperative astigmatism that was included was 0-1.25D. Cases with pre-existing ocular pathology were excluded, as were patients with incomplete postoperative data. We excluded 2 eyes that underwent mini-monovision surgery as these had slightly myopic targets. Our binocular data represents patients with bilateral Symfony IOL insertion reading binocularly.

## 3 RESULTS

A total of 94 eyes underwent Symfony IOL insertion from a cohort of 53 patients with 40 patients receiving bilateral Symfony IOLs. Forty-nine cases were cataract extractions and the remaining 54 were clear lens extractions. There was an even male-female split and more hyperopic than myopic patients (69 and 24 respectively).

There was an overall average postoperative monocular UDVA of 0.12 LogMAR (CI 0.09 - 0.14) and a binocular UDVA of 0.05 LogMAR (CI 0.12 - 0.09). The average monocular UNVA was 0.34 LogMAR (CI 0.32 - 0.35) was and the binocular UNVA was 0.29 (CI 0.27 - 0.31) LogMAR. The clear lens exchange group had a preoperative monocular average CDVA of 0.04 LogMAR (CI 0.03 - 0.06), a postoperative UDVA of 0.11 LogMAR (CI 0.09 - 0.14) and a UNVA of 0.33 LogMAR (CI 0.30 - 0.35). The cataract group had a preoperative monocular average CDVA of 0.35 LogMAR (CI 0.29 - 0.39) with a postoperative UDVA of 0.14 (CI 0.11 - 0.18) and a UNVA of 0.35 LogMAR (CI 0.32 - 0.38) (Table **1**). Comparing the cataract and CLE groups we found no difference in UDVA or UNVA (identical *P* values = 0.36). Our binocular data is presented in Table **2**.

**Fig. (1):**
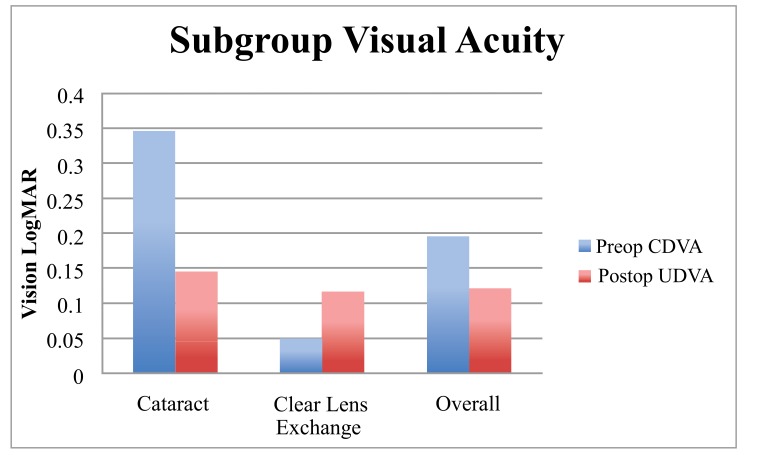
Postoperative uncorrected monocular distance visual acuity in subgroups based on preoperative corneal astigmatism in dioptres.

**Fig. (2):**
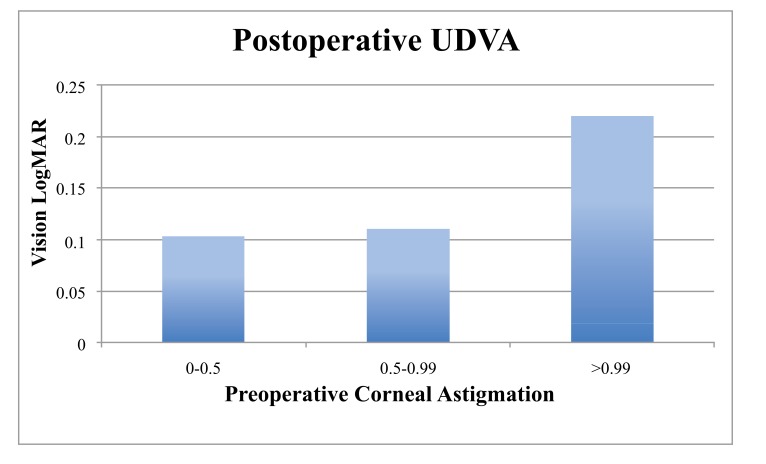
Postoperative uncorrected monocular distance visual acuity in cases with preoperative corneal astigmatism > 1D in magnitude.

**Fig. (3):**
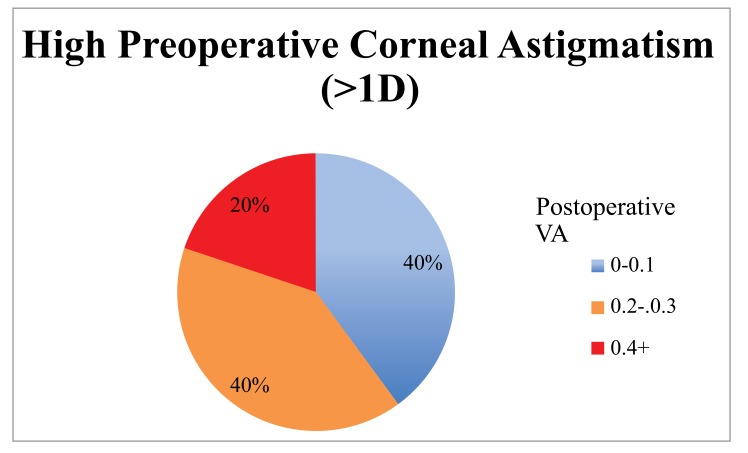
Postoperative uncorrected monocular distance visual acuity in cases with preoperative corneal astigmatism < 1D in magnitude.

When the cohort was divided into 2 groups based on the magnitude of preoperative corneal astigmatism (low 0 - 0.99 D, n = 80; high >1 D, n = 14) the low astigmatism group achieved statistically significantly better results (Table **1**, Figs. **1**, **2**, **3**). The low astigmatism group achieved a UDVA of 0.11 (6/7.5) LogMAR (CI 0.085 - 0.124) and the high astigmatism group 0.21 (6/9.5) LogMAR (CI 0.129 - 0.285) (*p*=0.0151). We can see in Figs. (**2** and **3**) that, as well as having an average postoperative UDVA one Snellen line lower than the low astigmatism group, the high astigmatism group also had a higher likelihood of a poor (> 6/15) visual outcome (20%* vs. *2%) (Figs. **2** and **3**).

**Fig. (4):**
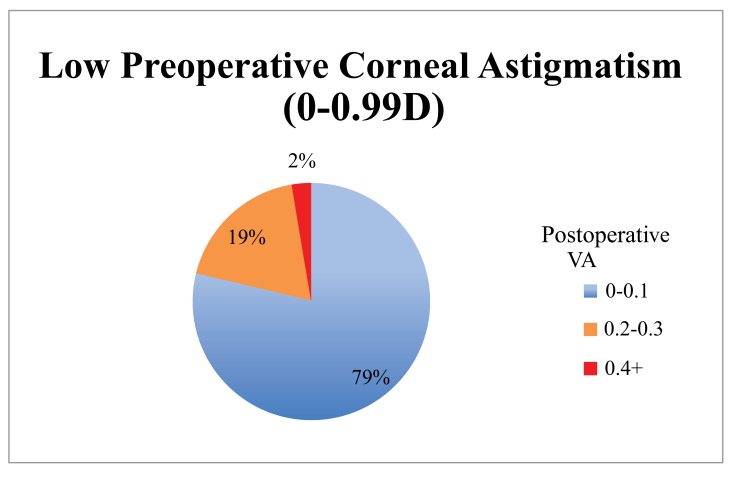
Postoperative monocular near visual acuity in cases with preoperative corneal astigmatism <1D in magnitude.

**Fig. (5):**
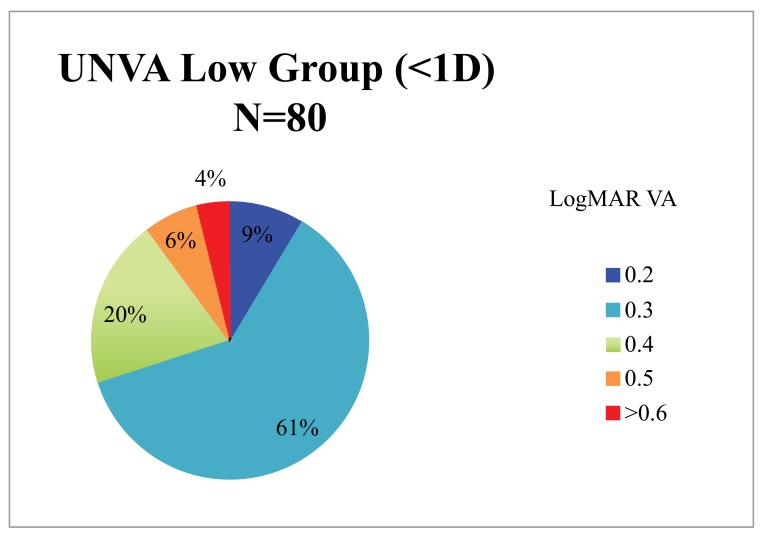
Postoperative monocular near visual acuity in cases with preoperative corneal astigmatism >1D in magnitude.

The low astigmatism group also had statistically significantly better UNVA than the high astigmatism group 0.33 LogMAR (CI 0.32 - 0.36)* vs. *0.39 LogMAR (CI 0.34 - 0.43) (*p* =0.034, CI 95%). The group with the higher levels of preoperative astigmatism had inferior and more unpredictable results than the low astigmatism group (Figs. **4** and **5**). A much smaller proportion achieved a UNVA of 0.20-0.30 LogMAR (N5-6) with just 36% of the high astigmatism group, compared with 70% of the low astigmatism group.

Cases with postoperative corneal astigmatism <1D in magnitude had better postoperative UDVA than those with >1D but these results did not reach statistical significance (0.11 [6/7.5^+1^] LogMAR* vs. *0.16 [6/7.5^+3^] LogMAR respectively, *p* = 0.238). There was no difference in visual outcomes or surgically induced astigmatism between patients who had clear corneal temporal incisions* vs. *those who had clear corneal located on the steep axis (0.12 [6/7.5^+1^]* vs. *0.12 [6/7.5^+1^] LogMAR respectively, *p* = 0.64) (0.43D* vs. *0.55D respectively, p = 0.5).

## 4 DISCUSSION

The Symfony IOL has been shown to be a suitable option for both cataract surgery and clear lens exchange in presbyopic patients. An expectation of complete independence from spectacles adds greater pressure to the surgeon to achieve the optimum refractive outcome. Pre-existing corneal astigmatism has a critical impact on visual results. Whilst patients are counselled that the lens may not result in spectacle independence, the expectation often remains that they will achieve this goal. We wished to evaluate the impact of preoperative astigmatism on visual outcomes in patients undergoing cataract surgery or clear lens extraction with an EDF IOL.

In our study population of 53 patients, the resulting average monocular postoperative UDVA of 0.12 LogMAR and UNVA of 0.34 LogMAR demonstrate the effectiveness of the Symfony IOL in addressing distance visual acuity whilst also providing effective near visual acuity. The results for binocular vision (n=40 patients) are comparable with other studies with an average binocular UDVA of 0.05 LogMAR and UNVA of N 6.05 (0.31 LogMAR) (Table **2**) [7].

Our results show that high levels of preoperative corneal astigmatism are associated with inferior results (Table **1**, Figs. **1**-**5**). The average monocular UDVAs and UNVAs were inferior and the visual results were more unpredictable in patients with high levels of preoperative corneal astigmatism. Patients with preoperative corneal astigmatism less than 1D were 39% more likely to achieve a UDVA of 0-0.10 LogMAR (6/6-6/7.5) and 34% more likely to achieve a UNVA of 0.20-0.30 LogMAR (N5-6) than those with a preoperative corneal astigmatism more than 1D. The group with high preoperative astigmatism also had a higher proportion of poor visual outcomes for both distance and near. Twenty percent of this group had monocular vision worse than LogMAR 0.4 (6/15), a level that would require spectacles whilst driving at a minimum. Given the levels of patient expectation associated with this IOL (and other premium IOLs) these results are especially important to avoid.

Moderate to high astigmatism is very prevalent in the general population - a study of 23,000 eyes found 1D or greater corneal astigmatism in one third of subjects [8]. Residual astigmatism has been shown to be a major source of dissatisfaction in patients with multifocal IOL insertion [9-11]. Options that attempt to address high levels of astigmatism include toric lenses, limbal relaxing incisions and laser refractive surgery.

Toric monofocal lenses are generally recommended for insertion in cases with astigmatism greater than 1.5D in magnitude. However, the superiority of toric lenses over monofocal IOLs has been demonstrated in patients with as low as 1D of corneal astigmatism [12]. Toric lenses offer the advantage of not requiring a separate procedure, carry no further risk and do not regress over time as other techniques can [13].

Previous studies have looked at visual outcomes in patients with the Symfony IOL. Monaco *et al*. and Sachdev *et al.* excluded patients with preoperative corneal astigmatism of greater than 0.75D [7, 14]. Cochener at al. excluded patients with postoperative corneal astigmatism of greater than 0.75D [15]. Pedrotti *et al.* compared a monofocal IOL with the Symfony IOL but did not correlate the level of preoperative astigmatism with visual results [16]. None of these studies evaluated the effect of preoperative astigmatism on visual results. Our study is the first to directly examine the effect of preoperative astigmatism on visual outcomes in an EDF IOL. We believe preoperative astigmatism to be a more practical surgical factor to analyse, compared with postoperative astigmatism, as it is a value available to the surgeon before the operation.

Previous authors have evaluated the effect of preoperative corneal astigmatism in patients with multifocal IOL insertion [17, 18]. Hayashi found that astigmatism had a greater blurring effect on multifocal IOLs than monofocal IOLs. This shows that astigmatism results in variable blurring may be lens dependent. Our study has attempted to evaluate the effect of astigmatism on EDF IOL visual outcome.

## CONCLUSION

Our study adds to the literature by demonstrating that patients with less than 1D of preoperative corneal astigmatism achieve excellent uncorrected near and distance acuity and have more predictable results. Patients with preoperative astigmatism greater than 1D are at significantly higher risk of having a poorer outcome. These patients may have better outcomes with toric EDF lenses. We would recommend careful discussion of visual outcomes in patients with preoperative corneal astigmatism of greater than 1D in order to manage their expectations.

## Figures and Tables

**Table 1:** Subgroup analysis of postoperative monocular logMAR uncorrected distance and near visual acuity (UDVA and UNVA; D= Dioptres, SD = Standard Deviation)

–	–	–	–	Preoperative Corneal Astigmatism		
–	Overall	CLE	Cataract	0-0.5D	0.5-0.99D	>1D
Total Eyes	94	45	49	60	20	14
UDVA	–	–	–	–	–	–
LogMAR	0.12	0.11	0.14	0.11	0.10	0.21
Mean ± SD	0.10	0.09	0.12	0.09	0.08	0.14
Median	0.10	0.10	0.10	0.10	0.10	0.20
Range	0-0.50	0-0.40	0-0.50	0-0.40	0-0.40	0-0.50
UNVA	–	–	–	–	–	–
LogMAR	0.34	0.33	0.35	0.33	0.35	0.39
Mean ± SD	0.09	0.07	0.10	0.09	0.10	0.07
Median	0.30	0.30	0.30	0.30	0.30	0.40
Range	0.20-0.65	0.20-0.50	0.20-0.65	0.20-0.65	0.30-0.65	0.30-0.50

**Table 2:** Uncorrected binocular LogMAR distance and near visual acuity (UDVA, UNVA) comparison between clear lens exchange (CLE) and cataract cases; SD = Standard Deviation.

–	Overall	CLE	Cataract
Total Patients	40	22	18
UDVA	–	–	–
LogMAR	0.05	0.05	0.05
Mean ± SD	0.07	0.07	0.08
Median	0	0.1	0
Range	-0.1-0.3	-0.1-0.2	0-0.3
UNVA	–	–	–
LogMAR	0.29	0.27	0.3
Mean ± SD	0.06	0.06	0.07
Median	0.3	0.3	0.3
Range	0.2-0.4	0.2-0.4	0.2-0.4

## References

[1] Luo B.P., Brown G.C., Luo S.C., Brown M.M. (2008). The quality of life associated with presbyopia.. Am. J. Ophthalmol..

[2] Law E.M., Aggarwal R.K., Kasaby H. (2014). Clinical outcomes with a new trifocal intraocular lens.. Eur. J. Ophthalmol..

[3] Sheppard A.L., Shah S., Bhatt U., Bhogal G., Wolffsohn J.S. (2013). Visual outcomes and subjective experience after bilateral implantation of a new diffractive trifocal intraocular lens.. J. Cataract Refract. Surg..

[4] Kamiya K., Hayashi K., Shimizu K., Negishi K., Sato M., Bissen-Miyajima H. (2014). Multifocal intraocular lens explantation: A case series of 50 eyes.. Am. J. Ophthalmol..

[5] Ito M., Shimizu K., Niida T., Amano R., Ishikawa H. (2014). Binocular function in patients with pseudophakic monovision.. J. Cataract Refract. Surg..

[6] Hamid A., Sokwala A. (2016). A more natural way of seeing: Visual performance of three presbyopia correcting intraocular lenses.. Open J. Ophthalmol..

[7] Monaco G., Gari M., Di Censo F., Poscia A., Ruggi G., Scialdone A. (2017). Visual performance after bilateral implantation of 2 new presbyopia-correcting intraocular lenses: Trifocal versus extended range of vision.. J. Cataract Refract. Surg..

[8] Hoffmann P.C., Hütz W.W. (2010). Analysis of biometry and prevalence data for corneal astigmatism in 23,239 eyes.. J. Cataract Refract. Surg..

[9] de Vries N.E., Webers C.A., Touwslager W.R., Bauer N.J., de Brabander J., Berendschot T.T., Nuijts R.M. (2011). Dissatisfaction after implantation of multifocal intraocular lenses.. J. Cataract Refract. Surg..

[10] Gibbons A., Ali T.K., Waren D.P., Donaldson K.E. (2016). Causes and correction of dissatisfaction after implantation of presbyopia-correcting intraocular lenses.. Clin. Ophthalmol..

[11] Dick H.B., Krummenauer F., Schwenn O., Krist R., Pfeiffer N. (1999). Objective and subjective evaluation of photic phenomena after monofocal and multifocal intraocular lens implantation.. Ophthalmology.

[12] Statham M., Apel A., Stephensen D. (2009). Comparison of the AcrySof SA60 spherical intraocular lens and the AcrySof Toric SN60T3 intraocular lens outcomes in patients with low amounts of corneal astigmatism.. Clin. Experiment. Ophthalmol..

[13] Rubenstein J.B., Raciti M. (2013). Approaches to corneal astigmatism in cataract surgery.. Curr. Opin. Ophthalmol..

[14] Sachdev G.S., Ramamurthy S., Sharma U., Dandapani R. (2018). Visual outcomes of patients bilaterally implanted with the extended range of vision intraocular lens: A prospective study.. Indian J. Ophthalmol..

[15] Cochener B., Concerto Study Group (2016). Clinical outcomes of a new extended range of vision intraocular lens: International multicenter concerto study.. J. Cataract Refract. Surg..

[16] Pedrotti E., Bruni E., Bonacci E., Badalamenti R., Mastropasqua R., Marchini G. (2016). Comparative analysis of the clinical outcomes with a monofocal and an extended range of vision intraocular lens.. J. Refract. Surg..

[17] Hayashi K., Manabe S., Yoshida M., Hayashi H. (2010). Effect of astigmatism on visual acuity in eyes with a diffractive multifocal intraocular lens.. J. Cataract Refract. Surg..

[18] Pepose J.S., Qazi A.M., Chu R., Stahl J. (2008). Maximizing satisfaction with presbyopia-correcting intraocular lenses: The missing links.. Am. J. Ophthalmol..

